# Endovascular Treatment of Pelvic Congestion Syndrome: Visual Analog Scale Follow-Up

**DOI:** 10.3389/fcvm.2021.751178

**Published:** 2021-11-17

**Authors:** Quentin Senechal, Perrine Echegut, Marine Bravetti, Marie Florin, Lamia Jarboui, Mehdi Bouaboua, Christophe Teriitehau, Jacques Feignoux, Francois Legou, Eric Pessis

**Affiliations:** Centre Cardiologique du Nord, Saint-Denis, France

**Keywords:** pelvic congestion syndrome, chronic pelvic pain, pelvic vein insufficiency, embolization, pelvic venous disorders, chronic pelvis vein incompetence, nutcracker syndrome (NCS), May–Thurner syndrome

## Abstract

**Purpose:** To evaluate medium-term clinical outcomes of transcatheter embolization and stenting in women with several pelvic venous disorders responsible for chronic pelvic pain and varicose veins of the lower limbs.

**Materials and Methods:** The study population included 327 consecutively recruited patients referred to the interventional radiology unit from January 2014 to December 2019 due to chronic pelvic congestion (91; 27.83%), lower limb varices (15; 4.59%), or a combination of both the symptoms (221; 67.58%). Preprocedural pelvic, transvaginal Doppler ultrasound (US), and MRI were conducted in all the patients and revealed anatomical varicosities and incompetent pelvic veins in 312 patients. In all the patients, selective catheterization demonstrated uterine venous engorgement, ovarian plexus congestion, or pelvic vein filling. Retrograde flow was detected on catheter venography in the left ovarian vein (250; 78%), the right ovarian vein (85; 26%), the left internal iliac vein (222; 68%), and the right internal iliac vein (185; 57%). Patients were followed-up at 1, 6, and 12 months, and years thereafter systematically by the referring angiologist and the interventional radiologist of center. They were contacted by telephone in November and December 2020 to assess pain perception and quality of life by using the visual analog scales from 0 to 10 with assessments made at the baseline and last follow-up. Of the 327 patients (mean age, 42 ± 12 years), 312 patients were suffering from pelvic congestion syndrome and 236 patients was suffering from lower limb varices. All underwent embolization by using ethylene vinyl alcohol copolymer (Onyx^®^). Eighty-five right ovarian veins, 249 left ovarian veins, 510 tributaries of the right internal iliac vein, and 624 tributaries of the left internal iliac vein were embolized. A cohort of patients also underwent nutcracker syndrome angioplasty (6.7%) and May–Thurner syndrome angioplasty (14%) with a stent placement.

**Results:** The initial technical success rate was 80.9% for embolization of pathological veins and 100% for stenting of stenoses. Overall, 307 patients attended 12-month follow-up visits and 288 (82%) patients completed the telephone survey at mean 39 (±18)-month postintervention. Main pelvic pain significantly improved from 6.9 (±2.4) pre- to 2.0 (±2.4) postembolization (*p* < 0.001), as did specific symptoms in each category. Improvement or disappearance of pain was achieved in 266/288 (92.36%) patients with improved quality of life in 276/288 (95.8%) patients. There were 16 minor and 4 major adverse events reported on the follow-up.

**Conclusion:** Pelvic vein embolization (Onyx^®^) is an effective and safe procedure with high clinical success and quality of life improvement rates.

## Introduction

Chronic pelvic pain (CPP) is a common health problem among women of childbearing age, which is responsible for about 10% of gynecological consultations (1). Several causes have been put forth including dilated pelvic veins, endometriosis, uterine leiomyomata, and malignant tumors. Taylor was the first to ascribe CPP symptoms to dilated pelvic veins and devised the term “pelvic congestion syndrome” (PCS) in 1949, but it is still poorly understood and underdiagnosed as being the culprit of CPP (2). While several pelvic venous disorders (PeVDs) definitions have been put forth, they all fundamentally encompass CPP as being linked to incompetent pelvic valves and structural vein abnormalities, causing gonadal vein reflux, iliac venous insufficiency, and pelvic venous engorgement (3). Both the clinical and radiological approaches that reveal pelvic vein incompetence are keys to the therapeutic workup. The prevalence of pelvic varices has been estimated at about 10% in the general female population, while up to 40% of them are likely to develop PeVD (4). This syndrome is characterized by CPP with chronic pelvic vein incompetence (CPVI), without any other identified cause, which persists for more than 6 months (5). The pain is commonly exacerbated by prolonged standing, prolonged sitting, at the end of the day, and in the perimenstrual period. Congestive dysmenorrhea, deep dyspareunia, and postcoital pain are common symptoms. Risk factors for PeVD include multiple pregnancies and hormonal influences (6).

Several therapeutic options have proven efficacious in alleviating pain and distress of patients including medical therapies such as medroxyprogesterone acetate, goserelin, and surgical interventions. As a curative-intent option, transcatheter embolization therapy has become a reference treatment in the PeVD setting (7). Following treatment of the bilateral gonadal and internal iliac veins by this modality, patients reported symptom improvement in 93–96% of cases with relatively good tolerability (8).

Nevertheless, published studies focused on interventional radiology in patients with PeVD are still scarce, while mostly including only small-sized samples with the short follow-ups. Ethylene vinyl alcohol copolymer (Onyx^®^), unlike sclerotherapy, allows for easy fluoroscopic monitoring and control with less risk than coils and glue of additional migration or recanalization. Consequently, this study sought to evaluate the clinical medium-term outcome regarding symptom relief and quality of life (QoL) based on the visual analog scale (VAS) scoring conducted before and after embolization by using ethylene vinyl alcohol copolymer in patients with PeVD.

## Materials and Methods

### Study Design

This retrospective study involved consecutively-recruited patients with PeVD suspected PCS or lower leg varicose (LLV) treated by using embolization from January 2014 to December 2019 at the interventional radiology unit. Patients were referred from gynecologists for PCS suspicion or from vascular surgeons and angiologists for concomitant or isolated varicosities of pelvic and lower extremity veins.

Inclusion criteria were CPP or LLV for more than 6 months with an imaging diagnosis of CPVI on Doppler ultrasound (US) and MRI. While the main concern for people with PeVD is CPP, varicose veins of the lower limbs of pelvic origin must be treated specifically.

Exclusion criteria involved other pelvic pathologies (compression of the pelvic veins by large fibroids, adenomyosis, salpingitis, and pelvic tumors) and pregnancy. It must be noted that endometriosis was not deemed an exclusion criterion provided that CPP had persisted for more than 6 months after appropriate medical endometriosis treatment with visualization of pelvic varices on imaging studies.

### Patient Population

**Figure 1:**
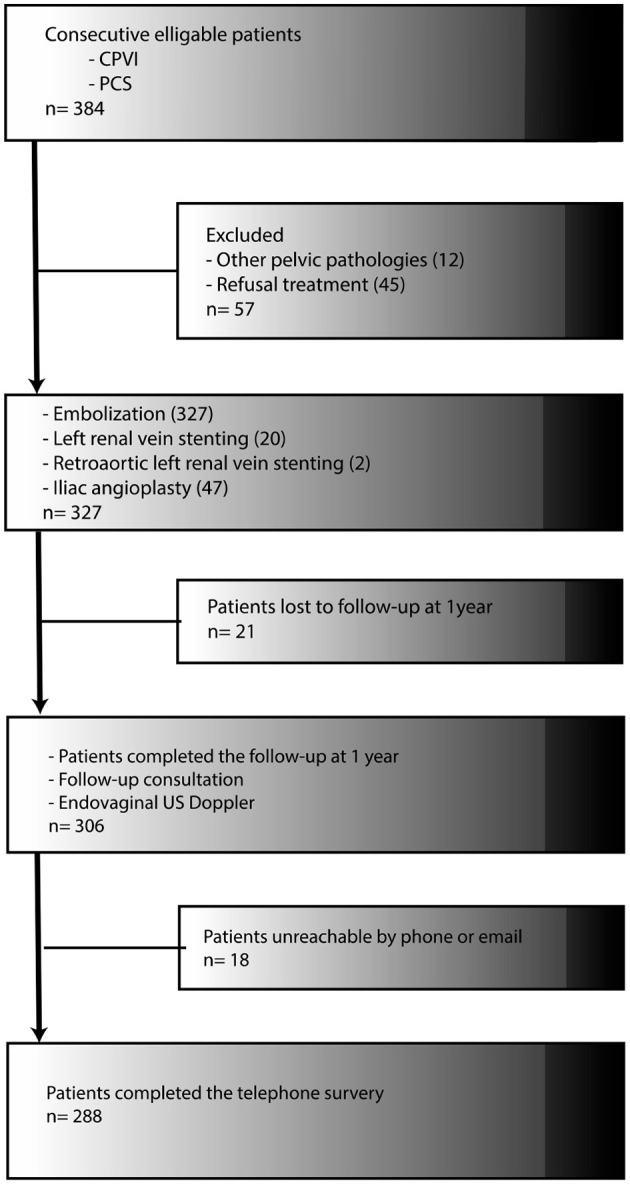
Trial enrollment and follow-up.

From January 2014 to December 2019, 384 patients were referred to the department for pelvic vein embolization in a CPVI setting. A total of 15 patients (3.9%) were referred for pelvic venous disorders at the origin of lower limb varices. All the other patients suffered from chronic non-cyclical pelvic pain that persisted for more than 6 months and all completed a pain assessment questionnaire. A total of 12 patients were excluded from analysis because of other pelvic pathologies, while another 45 patients did not undergo any embolization therapy for various reasons. Overall, 327 patients were considered for the analysis (Figure 1) and their demographic data have been summarized in Table 1.

**Table 1:** Patient baseline characteristics.

**Variable**	**Study cohort (***N*** = 327)**
Age (years), mean (SD), median [Q25–75], min–max	41.9 (11.6) 40.0 [33.0; 49.0] 16–69
Number of children (*n*), mean (SD), min–max	2.59 (1.63) 2.00 [2.0; 3.0] 0–10
Number of pregnancies (*n*), mean (SD), min–max	3.14 (2.05) 3.00 [2.0; 4.0] 0–11
Nulliparous, *n* (%)	39 (11.9)
**Endometriosis history**, ***n*** **(%)**	73 (22.32)
73 MRI or vaginal ultrasound: *n* positive (%)	58 (17.74)
27 Laparoscopy: *n* positive (%)	23 (7)
Not found	15 (4.6%)
Pelvic surgery history, *n* (%)	79 (24.16)
**Contraceptive treatment**, ***n*** **(%)**	
Combined oral contraceptives	37 (11.3)
Intrauterine device	30 (9.17)
Stopping ovulation and menstruation therapy	69 (21.1)
Trump ligature	3 (0.92)
None	188 (57.5)
**Menstruation**, ***n*** **(%)**	
Normal	162 (49.54)
Excessive bleeding	7 (2.14)
Shorter cycles	13 (3.98)
Period stop	63 (19.27)
Menopause	82 (25.01)
Medical history of stripping or endovenous thermal ablation	106 (32)
Medical history of sclerotherapy	85 (26)
Recurrence after surgery or sclerotherapy	110 (34)
**Embolization motive**, ***n*** **(%)**	
Pelvic congestion syndrome	312 (95.41)
Leg varicose veins	15 (4.59)

### Preinterventional Workup

All the patients benefited from a pretreatment consultation with clinical and radiological evaluation. During this visit, data on age, parity, prior LLV, past pelvic surgery, medical history, and associated pelvic pathologies were collected. All the patients were asked to subjectively assess the level of CPP, postcoital pain, lower limb pain, and QoL by using the VAS (9). The VAS score was rated from 0 to 10, where 0 represented “no pain” and 10 represented “worst pain possible.”

Other clinical symptoms that were assessed included pain on standing, pain on lying down, menstrual pain, and increased urinary frequency. At physical examination, lower limb, vaginal, and vulvar varicosities were assessed. The VAS assessments were made before embolization and during the follow-up.

All the patients benefited from pelvic, transvaginal, and lower limb Doppler US and pelvic MRI. In 127 cases, a CT angiogram of abdomen and pelvis was performed to identify anatomical variants and compression of the left renal veins and common iliac veins. Pelvic and transvaginal US were routinely conducted, with the patient in a supine or semi-upright position, by using transabdominal and transvaginal techniques with color flow and Doppler examinations in order to ascertain PeVD diagnosis according to the US criteria described by Park et al. (6). Reflux through the connections between the pelvis and lower extremity (escape points) has been documented with transperineal US. The inguinal (round ligament escape point), obturator, pudendal, superior, and inferior gluteal points can be responsible for varicose veins of the vulvar and lower extremity veins.

All the patients were initially examined with a routine MRI protocol for the pelvis that included the axial, coronal, and sagittal *T*2-weighted images, the axial *T*1-weighted images, and the contrast-enhanced *T*1-weighted images. Pelvic MR angiography was performed in coronal three-dimensional (3D) Dixon-Volumetric Interpolated Breathold Examination (VIBE) gadolinium sequences, so as to assess pelvic varicocele, diameter of dilated ovarian veins, pelvic differential diagnoses, and secondary congestion. The MRI criteria applied were the presence of at least four ipsilateral parauterine veins of varying caliber with at least one measuring more than 5 mm in diameter or an ovarian vein diameter exceeding 8 mm (10). During the pelvic MRI, we evaluated antegrade/retrograde flow of ovarian veins (left and right) on dynamic MRI. Patients who need a study of the details of anatomic structures, detecting, and staging vein stenosis were examined with a 256-detector CT scanner by using kV Assist (Apex GE Healthcare, Wisconsin, Boston, Massachusetts, USA). A bolus of 2.5 ml/kg of non-ionic contrast material was administered at a speed of 4 ml/s. Two scanning was delayed 40 and 90 s after injection of the iodinated contrast media and covered from the diaphragm to pelvis. Reconstruction included multiplanar reconstructions and volume rendering.

We used the PCS scoring system suggested by Champaneria et al. (11), which proposes a scoring model for reporting standards for clinical research studies evaluating diagnostic tests for PCS. The suggested diagnostic and scoring criteria were: presence of CPP (score 0 for <3 months, score 1 for 3–6 months, and score 3 for ≥6 months); presence of each of dysmenorrhea (= 1), dyspareunia (= 1), and pain after prolonged periods of standing (= 2); presence and location of visible varices in lower leg (= 1), upper thigh, buttocks, or vulva (= 1); presence of pelvic vein variants (= 2); diameter of ovarian veins at widest caliber (score 0 for diameter <4 mm, score 1 for diameter 4–5 mm, and score 3 for diameter >5 mm); presence of retrograde flow on Valsalva maneuver (= 2) refilling time ≥20 s (= 1); and presence of contralateral filling (= 2). The score out of a maximum of 20 gave a diagnosis of PCS as unlikely if <10, moderately likely if between 10 and 14, and highly likely if ≥15.

Patients with CPP and a high degree of PeVD suspicion underwent venographic assessment for diagnosing pelvic congestion with subsequent therapeutic embolization, as indicated. Significant stenosis of the left Common Iliac Vein (CIV) was narrowing of the lumen of the vessel by >50%, presence of prestenotic venous dilation in CT scan, reflux of contrast agent into the internal iliac vein, and contrasting of collateral pelvic veins. Symptomatic obstruction of the left renal vein (LRV) was defined by hematuria, flank pain, CT parameters, such as a beak sign, LRV diameter ratio, and pressure gradient between the LRV and inferior vena cava ≥3 mm Hg after left ovarian vein embolization.

Following the intervention, patients were followed-up by the referring angiologist and the interventional radiologist of center at 1, 6, and 12 months and yearly thereafter including clinical evaluation, pain and symptom assessment, QoL scoring, and Doppler US. In the event of persistent pain in the 6 months after the procedure, an indexed procedure with another venogram was carried out to reveal any pelvic vein reflux or other vein abnormalities.

### Embolization Technique

Intravascular embolization was intended to occlude ovarian or pelvic veins that presented spontaneous or induced reflux or other vascular abnormalities; always starting with the most pathological vein (most dilated veins with the most significant retrograde flow or the most distal ones).

Because of the veins of the uterus, ovaries, bladder, distal colon, and the parietal structures of the pelvis communicated, to each other and through the pelvic floor, with veins from the anterior thigh, perineum, buttock, and posterior thigh, we realized distal supraselective embolization of them when a retrograde flow was demonstrated. The specific pelvic veins embolized were based on the assessment of reflux and leakage from the pelvis to the lower limbs. All the embolization procedures were performed by two radiologists both presenting over 10 years of experience in interventional radiology. In all the cases, diagnostic venography was conducted by using a transfemoral or brachial approach under local anesthesia in semi-upright position. The brachial approach was used when the right ovarian vein had to be embolized. A 5-Fr introducer sheath (Radiofocus, Terumo Europe, Leuven, Belgium, UK) was placed in the femoral vein (or brachial vein) and a 4-Fr Cobra II customized guiding catheter (Terumo Europe, Leuven, Belgium, UK) was advanced up in order to investigate the gonadal veins and afferent vessels. For brachial access, a 4-Fr vertebral (Terumo Europe, Leuven, Belgium, UK) guiding catheter was used. A selective hand injection in each internal iliac vein was conducted, under a Valsalva maneuver, in order to detect venous incontinence and pelvic opacification. Thereafter, the catheter was advanced into each renal vein and pointing to the origin of the left ovarian vein within the left renal vein. Reflux into the left ovarian vein was investigated by injecting iodinated contrast medium into the left renal vein with reflux into right ovarian vein similarly investigated by selective iodinated contrast medium injection. An automated pump injector was used for contrast injections into the common femoral vein and the left renal vein (10–15 ml at 5 ml/s). After ovarian vein insufficiency was confirmed, the catheter was further advanced to the pelvic level with a selective pelvic-centered, hand-injection performed. These maneuvers were aimed at detecting pelvic varicose veins or reflux in the lower limb or groin under Valsalva maneuver. Embolization was indicated for all the ovarian and pelvic veins demonstrating reflux, whether spontaneously or following a Valsalva maneuver (Supplementary Figures 1A,B).

A microcatheter (Rebar^®^, Medtronic, or Progreat^®^, Terumo, Tokyo, Japan) was employed to selectively catheterize the most distal pathological vein. Onyx^®^ is an elastic ethylene vinyl alcohol copolymer that is dissolved in dimethyl sulfoxide (DMSO) with micronized tantalum powder, thereby providing contrast for fluoroscopic visualization (Supplementary Figures 1C,D). All the embolization procedures were conducted under controlled with an anesthesiologist with general sedation at the time of DMSO injection. The aim of embolization was to fill as many pelvic varicose veins as possible with Onyx^®^ and to close venous communications and reflux veins. Embolizations were usually performed up to the distal ovarian vein to include the branch points of all the possible venous collaterals and up to the anastomoses of the internal iliac vein. Onyx^®^ 34L was slowly injected under fluoroscopic control on road mapping, so as to ensure full control and visualization to diminish the risk of non-target embolization (12). No coils or occlusion balloon was employed, when Onyx^®^ reflux arises near an anastomosis of the internal iliac vein or not target veins since the injection was stopping few seconds. The microcatheter was then pulled back and the injection resumed. After embolization of pelvic varicose veins, the insufficient vein was embolized with regular removal of the catheter. No coils were used to complete the embolization. Following the embolization procedure, another pelvic venogram was carried out in order to visualize complete or incomplete occlusion of pathological veins. For managing complex lesions with multiple feeder branches, several embolization sessions were required to attain complete occlusion of pathological veins.

There was a combined treatment strategy for patients who also underwent nutcracker syndrome (NCS) anterior angioplasty, May–Thurner syndrome (MTS) angioplasty, and NCS posterior angioplasty. Iliac or left renal angioplasty was indicated when increased venous pressures, resulting from proximal obstruction with reflux, induced symptoms related either to varicose veins or to increased venous pressure. The aim of the therapeutic interventions was directed toward decreasing the upstream venous pressure. The procedure was performed at the same time as the embolization if the stenosis was explored on CT and confirmed with angiography. If not, the patient was informed and a CT scan was scheduled. Then, a new intervention was organized if specific symptoms persisted and was classified in indexed intervention. In all the cases, venous self-expanding stents with diameter of 14–16 mm in diameter by 40–60 mm in length for left renal vein and 14–18 mm in diameter by 60–90 mm for common iliac vein after sizing on CT scan. In case of residual stenosis, balloon angioplasty was performed. After stenting, all the patients received antiplatelet drugs (clopidogrel 75 mg) for 3 months after intervention.

Patients with multiple venous refluxes with significant pelvic varicose veins and multiple communications to the pelvic floor and extrapelvic veins of the thigh were rescheduled directly after the procedure if embolization was incomplete. Repeat embolization excluded intentional staging of embolization for complex lesions.

Moreover, intravascular or surgical treatment of symptomatic varicose limb veins was carried out in additional, separate sessions. Patients were hospitalized 6–24 h postembolization in order to monitor pain and venous access. They were then discharged with a 5-day sick leave protocol for home pain management under nursing control. For the management of postoperative pain, opioids were administered if necessary.

### Clinical Follow-Up

All the patients were followed-up clinically at 1, 6, and 12 months and then annually for 5 years. The additional clinical symptom data presented here were collected *via* telephone calls that were made in November and December 2020 to all the patients having undergone intravascular embolization between January 2014 and December 2019 in the institution. During this telephonic conversation, patients were interrogated about pain, dyspareunia, dysmenorrhea, mictional urgency, and associated symptoms. All the patients were asked to subjectively assess pain, symptoms, and QoL (activity, energy, mood in the family, and at work) level using the VAS (9).

### Study Endpoints

#### Visual Analog Scale Endpoints

Primary study endpoint was the assessment of pain improvement (pelvic, limb, and menstrual pain, dyspareunia, and mictional urgency) and QoL from baseline to the last follow-up.

#### Clinical Endpoints

Clinical success was defined as improvement or disappearance of pain. For better analysis of the results, the VAS pain scores were classified into four categories: no pain heaviness (0–1), mild pain (2–4), moderate pain (5–7), and severe pain (8–10). Clinical success was considered complete when the VAS score decreased to 0–1; the latter was considered partial when the VAS score dropped to a lower category such as from severe to moderate. No change was recorded when VAS score stayed in the same category. Worsening was noted when the score escalated to a higher category. Recurrence was defined as the return of pain after 6 months of clinical improvement to the same basic before treatment.

#### Technical Endpoints

Secondary endpoints were perioperative safety issues and postoperative complications. Complete technical success was defined as the occlusion of ovarian and iliac pelvic veins that previously displayed reflux or other vascular abnormalities on fluoroscopy and endovaginal Doppler US.

Complications were assessed according to the Society of Interventional Radiology grade reporting standards (13).

### Statistical Analysis

Qualitative variables were expressed as frequencies and quantitative variables were expressed as mean ± SD. Comparisons of quantitative variables were performed by using the Kruskal–Wallis testing with the Fisher's exact test applied for comparing qualitative variables. The outcomes of the hypothesis tests were two-tailed, with *p*-values inferior to 0.05 indicating statistical significance. The results of the satisfaction surveys were compared by using the Student's *t*-test. Analysis was performed by using the Microsoft^®^ (Issy-les-moulineaux, France) Office Excel or the Medistica^®^ (Paris, France) PValue.io software.

### Ethics

Before initiation of study, the Ethics Committee for Research in Imaging (CERIM) provided approval of the study protocol. Written informed consent was obtained from each patient prior to conducting any interventional procedure.

## Results

### Population Characteristics

The primary indication for embolization was PCS in 312 (95%) and LVV in 15 (5%) patients (Table 1).

### Pelvic Congestion Syndrome Diagnosis

The clinical examination, pelvic and transvaginal Doppler US, and pelvic MRI were instrumental in confirming PCS diagnosis in 315 (96.3%) patients (Table 2). Mean PCS symptom duration was 98 ± 108 months with the mean VAS-recorded pain intensity of 7.0 ± 2.4.

**Table 2:** Pelvic congestion syndrome.

**Variable**	**Study cohort (*N* = 327)**
Physical symptoms	
CPP, *n* (%)	315 (96.3)
CPP duration (Month), mean (SD), median [Q25–75], min–max	98.3 (108), 60.0 [24.0; 120], 0–650
Pelvic pain VAS, Mean (SD), Median [Q25–75], min–max	6.96 (2.43), 8.0 [5.0; 9.0], 0–10
Lateralized	93 (28.4)
Right	38 (12)
Left	55 (17)
Bilateral	234 (72)
Dysmenorrhea, *n* (%)	260 (79.5)
Dyspareunia, *n* (%)	164 (50.15)
Pain after intercourse, *n* (%)	226 (69)
Pain after intercourse, VAS, mean (SD), median [Q25–75], min–max	4.68 (3.51), 5.00 [0; 8.0], 0–10
Pain after prolonged standing, *n* (%)	274 (84)
Pain exacerbated in the evening	243 (74)
Dysuria or urinary urgency	176 (53.8)
Hemorrhoidal history	158 (48)
Left leg pain	4.39 (3.05) 5.00 [1.0; 7.0], 0–10
Right leg pain	4.17 (3.10) 4.00 [1.0; 7.0] 0–10
Visible varices, *n* (%)	
Lower legs	236 (72)
Vulva	35 (10.7)
Atypical varices in thigh or buttock	102 (31.2)
Imaging findings	
Retrograde flow on Valsalva maneuver*, *n* (%)	327 (100)
Maximum left ovarian vein diameter*, mean (SD)	7.05 (2.72) 8.0 [6.0; 9.0] 0–13
Maximum right ovarian vein diameter*, mean (SD)	4.14 (2.50) 4.0 [3.0; 5.0] 0–12
Retrograde flow**, *n* (%)	267 (81.7)
Pelvic vein dilatation**, *n* (%)	327 (100)
Maximum right ovarian vein diameter**, mean (SD) median [Q25–75], min–max (21 missing data [6.4%])	4.74 (2.76) 4.0 [3.0; 6.75] 0–15
Maximum left ovarian vein diameter**, mean (SD)	6.95 (2.78) 7.0 [5.0; 9.0] 0–15
Maximum right ovarian vein diameter***, mean (SD)	5.02 (2.91) 5.0 [3.0; 7.0] 0–15
Maximum left ovarian vein diameter***, mean (SD)	7.34 (3.15) 8.00 [5; 10] 0–15
Right internaliliacvein reflux***	185 (57%)
Leftinternaliliacvein reflux***	222 (68%)
Compression of left renal vein *, *n* (%)	73 (22.3)
Compression of left renal vein **, *n* (%)	58 (17.7)
Compression of left renal vein ***, *n* (%)	105 (32.1)
Compression of the left common vein *, *n* (%)	58 (18%)
Compression of the left common vein **, *n* (%)	57 (17.4)
Compression of the left common vein***, *n* (%)	127 (39%)
Pelvic vein variants, *n* (%)	76 (23)
Observation of filling on contralateral veins, *n* (%)	311 (95)
PCS score„ mean (SD), median [Q25–75], min–max *n* (%)	15.2 (2.21) 15.0 [14.0; 17.0] 6–18
Group A <10, *n* (%)	5 (1.53)
Group B 10–14, *n* (%)	99 (30.3)
Group C >14, *n* (%)	223 (68.2)

* *On Doppler ultrasound*.

** *On MRI*.

*** *Venography*.

*CPP, chronic pelvic pain; PCS, pelvic congestion syndrome; VAS, visual analog scale*.

### Venography Findings

The venograms performed demonstrated pelvic vein dilatation and retrograde venous flow under Valsalva maneuver in all 327 (100%) patients, while a retrograde venous flow without Valsalva maneuver was recorded in 267 (82%) patients. Concerning with the internal iliac veins, a right retrograde flow was noticed in 185 (57%) patients and a left retrograde flow was noticed in 222 (68%) patients. The mean PCS was retrospectively assessed by using the PCS scoring system suggested by Champaneria et al. (11) as follows: 0.8 ± 1 for group A (PCS unlikely), 6.29 ± 2.8 for group B (PCS moderately likely), and 7.39 ± 2.0 for group C (PCS highly likely).

### Embolization Results

Overall, 614 embolization procedures were performed in 327 patients on overall 1,468 veins, meaning that 1.9 (±0.9) procedures on 4.5 veins were performed per patient on average, including 249 (76%) left ovarian vein and 85 (26%) right ovarian vein, 510 tributaries of the right internal iliac vein (uterovaginal, internal pudendal, obturator, superior, and inferior gluteal veins), and 624 tributaries of the left internal iliac vein embolizations. Further therapeutic details have been provided in Table 3. A total of 69 angioplasties with stent placement (22 for NCS and 47 for MTS) were carried out.

**Table 3:** Treatment.

**Variable**	**Study cohort (*N* = 327)**
Number of procedures (614)	1.88 (0.93) 2.00 [1.0; 2.0] 1–5
Recurrence	14 (4.28)
NCS anterior angioplasty, *n* (%)	20 (6.1)
MTS angioplasty, *n* (%)	47 (14)
NCS posterior angioplasty, *n* (%)	2 (0.61)
**Embolization**, ***n*** **(%)**	
Right ovarian vein	85 (26)
Left ovarian vein	249 (76)
Right uterine vein	150 (46)
Left uterine vein	250 (78)
Right pudendal vein	199 (61)
Left pudendal vein	263 (80)
Right obturator vein	114 (35)
Left obturator vein	89 (27)
Right gluteal inferior vein	42 (13)
Left gluteal inferior vein	20 (6.1)
right gluteal superior vein	5 (1.5)
Left gluteal superior vein	2 (0.61)
Bilateral ovarian vein	67 (20.5)
Patient with ovarian vein	265 (81)
Embolization *n*, mean (SD)	614, 1.88 (±0.93)
**Technical success**	
Occluded vein, *n* (%), per person	1,468 (80.9) 4.5
Stent deployment *n* (%)	69 (100)

*NCS, nutcracker syndrome; MTS, May–Thurner syndrome; VAS, visual analog scale*.

A total of 14 patients (4.6%) required repeated treatment in an already embolized vein because of symptom recurrence. A technical embolization failure occurred in one patient involving an ovarian vein with an abundant collateral system. It must be noted that LVV was generally treated in subsequent, separate sessions.

There were 20 complications recorded postoperatively including four major complications as follows: an allergic bronchospasm requiring 72-h hospitalization, a false common femoral artery aneurysm treated by direct embolization, one asymptomatic Onyx^®^ migration, and one infectious syndrome at day 15 postembolization. There were 16 minor complications recorded as follows: a panic attack, seven delayed allergies, a severe vagal reaction requiring 1-day hospitalization, four phlebitis at perfusion site cases, and four dyspnea cases at home, yet without any abnormality detected upon checkup in the emergency room. One patient suffering from salpingitis was operated at 1-month postintervention. During the overall follow-up, no long-term complications were reported.

### Follow-Up

The data retrieved at the 1-year consultation regarding symptom relief are given in Table 4. Of the 327 initial patients, 306 patients completed the questionnaire at 1-year follow-up visit. Pelvic pain was still present in 114 (37%) patients, but its mean VAS-recorded intensity had decreased from 7.0 (±2.4) before treatment to 1.2 (±1.9) at 1 year (*p* < 0.001). Dysmenorrhea, dyspareunia, and postcoital pain that had been present in 260 (±79%), 164 (±50%), 226 (±69%) patients prior to embolization were only still present in 58 (±19%), 57 (±19%), and 34 (±11%) patients at 1 year with markedly decreased intensity levels (*p* < 0.001).

**Table 4:** Symptom outcomes at 12-month consultation.

**Variable**	**Study cohort (*N* = 327)**
Follow-up at 12 month: lost patients in numbers (%)	21 (6.4)
**Variable**	**Study cohort (*****N*** **= 306)**
Pelvic pain, *n* (%)	114 (37.25)
Pelvic pain VAS, mean (SD)	1.23 (1.90) 0 [0; 3.0] 0–8
Dysmenorrhea, *n* (%)	58 (19)
Dyspareunia, *n* (%)	57 (18.6)
Pain after intercourse, *n* (%)	34 (11.1)
Pain after intercourse, VAS, mean (SD)	0.508 (1.46) 0 [0; 0] 0–8
Pain after prolonged standing, *n* (%)	25 (8.2)
Pain exacerbated in the evening	24 (7.9)
Dysuria	13 (4.25)
Left leg pain	0.840 (1.49) 0 [0; 1.0] 0–8
Right leg pain	0.931 (1.60) 0 [0; 1.0] 0–8
Visible varices	174 (57)

The US data retrieved at 1-year consultation in comparison with the preembolization results are given in Table 5. Of the 327 initial patients, 307 patients underwent US investigation at 1-year consultation. As can be seen in Table 6, statistically significant improvements (*p* < 0.001) were recorded on all the US parameters.

**Table 5:** Symptom outcomes at last telephone follow-up.

**Variable**	**Study cohort (*N* = 288)**
Follow-up (months), mean (SD)	38.9 (18.5) 25 [13.0; 39.0] 6–82
Pelvic pain VAS, mean (SD)	2.04 (2.45) 1.00 [0; 4.0], 0–9
Dysmenorrhea, *n* (%)	84 (29, 2)
Dyspareunia, *n* (%)	54 (18.75)
Pain after intercourse VAS, mean (SD)	0.833 (1.97) 0 [0; 0] 0–10
Pain after prolonged standing, *n* (%)	22 (7.3)
Pain left leg	1.86 (2.48) 1.00 [0; 4.0] 0–10
Pain right leg	1.84 (2.38) 0 [0; 3.25] 0–9
Visible varices	176 (61.1)
Improvement in quality of life	6.77 (2.38) 7.00 [5.0; 8.0] 0–10
VAS, Mean (SD)	276 (95.8)
*n* (%), <2	13 (4.5)
[2–4]	15 (5.2)
[4–6]	51 (17.7)
[6–8]	73 (25.3)
≥8	132 (45.8)
Post-embolization pregnancy	17 (5.9)

*VAS, visual analog scale*.

**Table 6:** Ultrasound results at 12-month consultation.

	**Pre-embolization** **(***n*** = 327)**	**Post-embolization** **(***n*** = 307)**	***p*** **-value**	**Test**
MTS Doppler, *n* (%)	58 (18)	5 (1.6)	<0.001	McNemar
NCS Doppler, *n* (%)	73 (22)	6 (2)	<0.001	McNemar
Right ovarian vein reflux	37 (11)	3 (0.98)	<0.001	McNemar
Left ovarian vein reflux	210 (64)	3 (0.98)	<0.001	McNemar
Leak points/ patient, *n* (SD)	1.38 (±1.39)	0.312 (±0.65)	<0.001	Paired *t*-test
Right pudendal leakage	139 (43)	33 (11)	<0.001	McNemar's
Left pudendal leakage	122 (37)	25 (8.1)	<0.001	McNemar
Right ovarian leakage	20 (6.1)	2 (0.65)	<0.001	McNemar
Left ovarian leakage	15 (4.6)	4 (1.3)	<0.001	McNemar
Right iliac leakage	41 (13)	11 (3.6)	<0.001	McNemar
Left iliac leakage	44 (13)	8 (2.6)	<0.001	McNemar
Right gluteal inferior leakage	12 (3.7)	2 (0.65)	<0.001	McNemar
Left gluteal inferior leakage	17 (5.2)	1 (0.333)	<0.001	McNemar
Right gluteal superior leakage	17 (5.2)	2 (0.65)	<0.001	McNemar
Left gluteal superior leakage	13 (4)	4 (1.3)	<0.001	McNemar
Right clitoral leakage point	6 (1.8)	3 (0.98)	<0.001	McNemar
Left clitoral leakage point	5 (1.5)	1 (0.33)	<0.001	McNemar
Right saphenous vein insufficiency			<0.001	McNemar's Chi-squared test
None	216 (66)	271 (88)		
Long saphenous	94 (29)	28 (9.1)		
Short saphenous	13 (4)	8 (2.6)		
Both	4 (1.2)	0 (0)		
Left saphenous vein insufficiency			Nan	McNemar's Chi-squared test
None	215 (66)	268 (87)		
Long saphenous	87 (27)	29 (9.4)		
Short saphenous	21 (6.4)	9 (2.9)		
Both	4 (1.2)	1 (0.33)		
Left saphenous vein insufficiency, *n* (%)	112 (34)	39 (13)	<0.001	McNemar
Pelvic vein insufficiency, *n* (%)	327 (100)	48 (15.6%)	<0.001	McNemar

*MTS, May–Thurner syndrome; NCS, nutcracker syndrome*.

Of the treated patients, 288 patients completed the telephone survey at a mean 39 (±18.5) months follow-up with outcome data compared to pre-embolization ones, as listed in Table 7. As can be seen, the mean pelvic pain level had significantly improved from 7.0 (±2.4) to 2.0 (±2.4) after embolization (*p* < 0.001). Improvement or disappearance of pain was achieved in 266/288 (92.3%) patients. Complete clinical success was recorded in 155 (53.8%) cases and partial success was recorded in 101 (35%) cases. No clinical change was observed in 29 (10.1%) cases. Worsening was noted in three patients (1.0%). One patient reported a deterioration in her QoL due to vulvar pain during sexual intercourse and sports and two others suffering from enhanced inguinal pain. Significant improvement (*p* < 0.001) in each specific symptom category was similarly reported with postcoital pain improving from 4.7 (±3.5) to 0.8 (±2.0), right inferior limb pain from 4.1 (±3.1) to 1.8 (±2.4), and left inferior limb pain from 4.4 (±3.0) to 1.8 (±2.4). Almost all the patients (276/288; 97.5%) reported an improvement in QoL by a mean of 6.7 (±2.4) with only seven patients (2.5%) declaring no amelioration.

**Table 7:** VAS-related symptom outcome at last telephone follow-up vs. baseline.

**Variable**	**Pre-embolization** **(***n*** = 327)**	**Post-embolization** **(***n*** = 288)**	**Δ mean**	***p*** **-value**	**Test**
VAS pelvic pain, mean (SD)	6.96 (±2.43)	2.04 (±2.45)	−4.92	<0.001	Paired *t*-test
VAS post coital, mean (SD)	4.68 (±3.51)	0.833 (±1.97)	−3.85	<0.001	Paired *t*-test
VAS pain right leg	4.17 (±3.10)	(±2.38)	−2.32	<0.001	Paired *t*-test
VAS pain left leg	4.39 (±3.05)	1.82 (±2.42)	−2.56	<0.001	Paired *t*-test
Dyspareunia, *n* (%)	164 (50)	54 (19)		<0.001	McNemar
Dysmenorrhea, *n* (%)	260 (80)	84 (29)		<0.001	McNemar
Visible varices *n* (%)	236 (72)	176 (61)		<0.001	McNemar

*VAS, visual analog scale*.

Overall, 14 (5%) patients experienced a recurrence of symptoms after clinical improvement. Statistical analysis found no significant difference in the follow-up according to treatment variables (bilateral vs. unilateral embolization with or without stenting).

A total of 17 (6%) patients carried a pregnancy to term. Overall, 259 patients (93%) had no change in menstrual periods, 8 (3%) patients experienced irregular cycles, and 21 (7%) patients had an improvement in menstrual cycle regularity after treatment.

## Discussion

Analysis of this large cohort of PCS women that underwent endovascular treatment revealed significant improvements in overall pain perception levels and each of the specific symptom categories and in QoL.

In this study, the imaging workup by using different techniques proved to be instrumentally useful. The transcatheter venography guided by MRI and Doppler US enabled to opacify major venous channels with their extensive venous tributary network including ovarian, uterine, vaginal, myometrial, and lower leg anastomoses. The CT scan has also allowed to better evaluate venous compressions and to detect the numerous anatomical variants that are sources of therapeutic errors. This combinative approach was actually effective for tackling impaired venous reflux. The disappearance of Doppler venous reflux following embolization testified to the success of intervention. In addition to investigation of abdominal US, an additional endovaginal investigation was carried out. This was effectively useful, as it enabled to further objectify pelvic reflux and measure the diameters of pathological veins as well. Likewise, these complementary imaging techniques permitted to better control the outcome of the intravascular intervention. Embolization was deemed complete when Doppler examination revealed no clinically relevant venous pelvic leaks into lower limb veins with a complete occlusion of pelvic varices and dilated veins. Notably, we would like to remind the reader that a proper control of the interventional outcome including transvaginal Doppler US is paramount.

In this study, pain improvement was 92.3% with a median follow-up period of 39 months, which is similar to the results of other studies. While the use of coils, with or without sclerotherapy, glue, and plugs has been most commonly reported for pelvic embolization, no study has reported significant differences in clinical outcomes between one specific embolization agent and another or in a combination of them. In 1993, Edward et al. reported the first case of uterine ovarian varices embolization (14). Since then, several other studies have been conducted with excellent results, but with fewer patients recruited than ours (15–18). Kim et al. reported on 131 patients that were treated with embolization by using sodium morrhuate and Gelfoam (16). Laborda et al. reported the long-term follow-up data of 202 patients with PCS that underwent coil embolization (17). De Gregorio et al. reported data of 520 patients that underwent coil or plug embolization of the four main pelvic veins (19). In contrast to this study, patients with PeVD associated with vascular compression syndromes were excluded. The VAS result and recurrence were similar with 57 minor complications (10.9%), 11 major complications (2.1%), and with 7 cases (1.34%) of device migration to the lung. Marcelin et al. (20) reported a clinical efficacy of 94.1% with a median follow-up of 22 months with Onyx^®^.

Major differences between the published studies and ours consist of the embolization agent used, potential stent deployment for clinically relevant venous obstacles applied, and our avoidance to systematically treat venous parts without relevant reflux. Our decision to employ Onyx^®^ as an embolizing agent was based on several reasons that we carefully evaluated. Yet, in our view, several shortcomings such as clinical failures, coil migration, and recurrences were reported following coil embolization. We suggest that it is necessary to treat all the pathological veins, as necessary, with subsequent control imaging. Moreover, ovarian and iliac coils embolization lead to a risk of difficulty in treating a clinical recurrence. With all this taken together, we eventually selected another embolic agent: ethylene vinyl alcohol copolymer (Supplementary Figure 1).

Onyx^®^ is available in two different viscosities with higher viscosity offering better control upon the embolic agent being injected. Instead, a lower viscosity formulation may be preferred when the target site is distant from the microcatheter tip with the liquid embolic agent enabled to flow deeper. The viscosity of Onyx^®^ used on these patients was determined on a case-by-case basis according to the risk of migration.

This technique, thus, permits a controlled and secure administration. Onyx^®^ has been successfully applied in the treatment of various vascular lesions including neurovascular malformations, aneurysm repair, visceral aneurysm, and PCS with positive results and low complication rates reported (12, 20). It must be mentioned that a major disadvantage of using Onyx^®^ technique is its high price. Another disadvantage is the pain during injection of DMSO requiring the intensification of anesthesia. This must, however, be weighed against the overall practicability of the method with its reduced complication, recurrence, and reintervention rates.

Based on this experience and owing to an improved understanding of the underlying pathology, we recommend investigating and treating the entire pathological vein network. If the treatment of venous compressions included in the series can lead to confusion on the interpretation of the results, it seemed important to us to report the results in accordance with the international recommendations (7, 21). Venous disorders of the pelvis are associated with a spectrum of symptoms arising from both the refluxes, most commonly involving the gonadal and internal iliac veins and obstruction, usually of the left renal and iliac veins. Primary obstruction of the left renal or either common iliac veins can produce secondary reflux in the left ovarian or either internal iliac veins and be responsible for clinical failure or recurrence in case of embolization without specific treatment of the stenosis. No statistically significant difference was found after treatment when dissociating patients who had or had not received a stent. However, there were more nulliparous patients in the population with a nutcracker (35–10%; *p* < 0.01) and more dyspareunia (44–17%; *p* < 0.01) compared to all the patients without a nutcracker. Similarly, there was no significant statistical difference between the population that underwent unilateral or bilateral embolization of the ovarian veins after treatment. However, there was more pain after intercourse (79–67%; *p* = 0.047), dysmenorrhea (88–77%; *p* = 0.052), greater intensity of pain in the right lower limb (4.99 ± 3.29 vs. 3.96 ± 3.02; *p* = 0.023), and pelvic pain (7.36 ± 1.87 vs. 6.86 ± 2.55; *p* = 0.074) before treatment in the population that underwent bilateral embolization.

Indeed, significantly different results depending on treatment completeness. In this study, the VAS-recorded symptom improvement was the best in patients that underwent complete therapy amounting to 7.0 ± 2.3. In contrast, for patients with persistent pelvic varices, it was only 5.0 ± 2.0. For complete treatment, several treatment sessions may be required some weeks apart with a full diagnostic workup to be performed prior to each new intervention.

Dysmenorrhea, dyspareunia, and inferior limb varices that had been present in 260, 164, and 226 patients, respectively, prior to embolotherapy were still present in only 84, 54 and 176 patients, yet with markedly decreased intensity levels.

According to the study by Kim et al. (16), ovarian function was not investigated in this study. Given the fact that 17 women (6%) became pregnant during the follow-up and delivered full-term babies, in addition to the absence of major menstrual cycle-related modifications, it can be assumed that pelvic vein embolization had no deleterious impact on ovarian function.

The small statistically significant increase in pain at the end of this study compared to the level of pain at the 1-year consultation, which was not reported from other studies, can be explained by the strategy of stopping analgesics after stabilization for all the patients and continuous hormonal treatments in agreement with the referring gynecologist to the patient.

It was important to note that no statistical difference was found in the evaluation of pain and also QoL scores according to the presence or absence of endometriosis.

Several limitations of this study must be highlighted. The major limitation of study is its retrospective, non-randomized study design, with the lack of a control group. Secondly, US evaluation criteria were not predefined prior to the study initiation, which may have contributed to some inconsistency in US assessments. We used the VAS scale to evaluate QoL, an applicable questionnaire of QoL assessments would have been more relevant. Lastly, of the 327 patients, only 288 patients attended the last follow-up telephonic interview. This may have somewhat biased the outcome data. Prospective randomized studies are needed to confirm these encouraging results.

In conclusion, embolization of pelvic veins by using the Onyx^®^ technique and stent placement has been shown to be an effective and safe technique, resulting in relevant clinical success, with an overall improvement of pain and other symptoms and QoL.

## Data Availability Statement

The original contributions presented in the study are included in the article/Supplementary Material, further inquiries can be directed to the corresponding author/s.

## Ethics Statement

The studies involving human participants were reviewed and approved by the Ethics Committee for Research in Imaging (CERIM). The patients/participants provided their written informed consent to participate in this study.

## Author Contributions

QS, PE, and MBr contributed to conception and design of the study. QS and MF performed the statistical analysis. MBo and LJ reviewed the MRI. FL and JF reviewed the CT Scan. EP wrote sections of the manuscript. All authors contributed to manuscript revision, read, and approved the submitted version.

## Conflict of Interest

The authors declare that the research was conducted in the absence of any commercial or financial relationships that could be construed as a potential conflict of interest.

## Publisher's Note

All claims expressed in this article are solely those of the authors and do not necessarily represent those of their affiliated organizations, or those of the publisher, the editors and the reviewers. Any product that may be evaluated in this article, or claim that may be made by its manufacturer, is not guaranteed or endorsed by the publisher.

## Supplementary Material

Supplementary Figure 1 Onyx^®^ embolization for recurrence of pelvic congestion syndrome (PCS). Patient referred to our institution for recurrence of PCS. The treatment had been performed in two stages in two different centers 3 years earlier. A first embolization of the uterovaginal varices and the left ovarian vein with coils and aetoxisclerol. The second embolization of uterovaginal, right uterovaginal, and a recurrence of the left ovarian vein with cyanoacrylate glue. **(A)** Venography demonstrated a recurrence of reflux of the left ovarian vein with numerous collaterals around the coils. This recurrence is responsible for pelvic venous insufficiency with ovarian varicose veins, dilated left uterine, and utero-ovarian plexuses with contralateral communication by dilated uterine and arcuate veins supplying the right uterine plexus, which is also dilated. **(B)** Valsalva maneuver shows bilateral uterovaginal reflux. **(C)** Onyx^®^ embolization. **(D)** Non-subtracted pelvic venography shows complete occlusion with Onyx^®^ in dilated left ovarian veins, left dilated utero-ovarian plexus, bilateral uterovaginal plexus, and arcuate uterine veins. *Black, Cyanoacrylate glue; *White, Coils; Arrows, Dilated veins of the utero-ovarian plexus; Double arrows, Dilated veins of the uterovaginal plexus; Arrows head, Onyx^®^ Double arrows head, Microcatheter.

## References

[1] Robinson JC. Chronic pelvic pain. Curr Opin Obstet Gynecol. (1993) 5:740–3. 10.1097/00001703-199312000-00005 8286684

[2] Taylor HC Jr. Vascular congestion and hyperemia; their effect on structure and function in the female reproductive system. Am J Obstet Gynecol. (1949) 57:211–30. 18107627 10.1016/0002-9378(49)90422-6

[3] Liddle AD Davies AH. Pelvic congestion syndrome: chronicpelvic pain caused by ovarian and internal iliac varices. Phlebology. (2007) 22:100–4. 10.1258/026835507780807248 18268860

[4] Belenky A Bartal G Atar E Cohen M Bachar GN. Ovarian varices in healthy female kidney donors: incidence, morbidity, and clinical outcome. Am J Roentgenol. (2002) 179:625–7. 10.2214/ajr.179.3.1790625 12185031

[5] Desimpelaere JH Seynaeve PC Hagers YM Appel BJ Mortelmans LL. Pelvic congestion syndrome: demonstration and diagnosis by helical CT. Abdom Imaging. (1999) 24:100–2. 10.1007/s002619900451 9933685

[6] Park SJ Lim JW Ko YT Lee DH Yoon Y Oh JH. Diagnosis of pelvic congestion syndrome using transabdominal and transvaginal sonography. Am J Roentgenol. (2004) 182:683–8. 10.2214/ajr.182.3.1820683 14975970

[7] Antignani PL Lazarashvili Z Monedero JL Ezpeleta SZ Whiteley MS Khilnani NM. Diagnosis and treatment of pelvic congestion syndrome: UIP consensus document. Int Angiol. (2019) 38:265–83. 10.23736/S0392-9590.19.04237-8 31345010

[8] Chung MH Huh CY. Comparison of treatments for pelvic congestion syndrome. Tohoku J Exp Med. (2003) 201:131–8. 10.1620/tjem.201.131 14649734

[9] Wewers ME Lowe NK. A critical review of visual analogue scales in the measurement of clinical phenomena. Res Nurs Health. (1990) 13:227–36. 10.1002/nur.4770130405 2197679

[10] Coakley FV Varghese SL Hricak H. CT and MRI of pelvic varices in women. J Comput Assist Tomogr. (1999) 23:429–34. 10.1097/00004728-199905000-00018 10348450

[11] Champaneria R Shah L Moss J Gupta JK Birch J Middleton LJ. The relationship between pelvic vein incompetence and chronic pelvic pain in women: systematic reviews of diagnosis and treatment effectiveness. Health Technol Assess. (2016) 20:1–108. 10.3310/hta20050 26789334 PMC4781546

[12] Guimaraes M Wooster M. Onyx (Ethylene-vinylAlcoholCopolymer) in peripheral applications. Semin Intervent Radiol. (2011) 28:350–6. 10.1055/s-0031-1284462 22942553 PMC3312165

[13] Black CM Thorpe K Venrbux A Kim HS Millward SF Clark TW. Research reporting standards for endovascular treatment of pelvic venous insufficiency. J Vasc Interv Radiol. (2010) 21:796–803. 10.1016/j.jvir.2010.02.017 20494288

[14] Edwards RD Robertson IR MacLean AB. Case report: pelvic pain syndrome—successful treatment of a case by ovarian vein embolization. Clin Radiol. (1993) 47:429–31. 10.1016/S0009-9260(05)81067-0 8519153

[15] Scultetus AH Villavicencio JL Gillespie DL Kao TC Rich NM. The pelvic venous syndromes: analysis of our experience with 57 patients. J Vasc Surg. (2002) 36:881–8. 10.1067/mva.2002.129114 12422096

[16] Kim HS Malhotra AD Rowe PC Lee JM Venbrux AC. Embolotherapy for pelvic congestion syndrome: long-term results. J Vasc Interv Radiol. (2006) 17(2 Pt 1):289–97. 10.1097/01.RVI.0000194870.11980.F8 16517774

[17] Laborda A Medrano J de Blas I Urtiaga I Carnevale FC de Gregorio MA. Endovascular treatment of pelvic congestion syndrome: visual analog scale (VAS) long-term follow-up clinical evaluation in 202 patients. Cardiovasc Intervent Radiol. (2013) 36:1006–14. 10.1007/s00270-013-0586-2 23456353

[18] Gandini R Chiocchi M Konda D Pampana E Fabiano S Simonetti G. Transcatheter foam sclerotherapy of symptomatic female varicocele with sodium-tetradecyl-sulfate foam. Cardiovasc Intervent Radiol. (2008) 31:778–84. 10.1007/s00270-007-9264-6 18172712

[19] De Gregorio MA Guirola JA Alvarez-Arranz E Sánchez-Ballestin M Urbano J Sierre S. Pelvic venous disorders in women due to pelvic varices: treatment by embolization: experience in 520 patients. J Vasc Interv Radiol. (2020) 31:1560–9. 10.1016/j.jvir.2020.06.017 32855049

[20] Marcelin C Izaaryene J Castelli M Barral PA Jacquier A Vidal V. Embolization of ovarian vein for pelvic congestion syndrome with ethylene vinyl alcohol copolymer (Onyx^®^). Diagn Interv Imaging. (2017) 98:843–8. 10.1016/j.diii.2017.05.011 28647478

[21] Meissner MH Khilnani NM Labropoulos N Gasparis AP Gibson K Greiner M. The symptoms-varices-pathophysiology classification of pelvic venous disorders: a report of the American Vein & Lymphatic Society international working group on pelvic venous disorders. J Vasc Surg Venous Lymphat Disord. (2021) 9:568–84. 10.1016/j.jvsv.2020.12.084 33529720

