# Supplementary figures and images for: Endovascular Treatment of Pelvic Congestion Syndrome: Visual Analog Scale Follow-Up
Source: Front Cardiovasc Med. 2021 Nov 17;8:751178. doi: 10.3389/fcvm.2021.751178 (PMC8635860; doi:10.3389/fcvm.2021.751178)

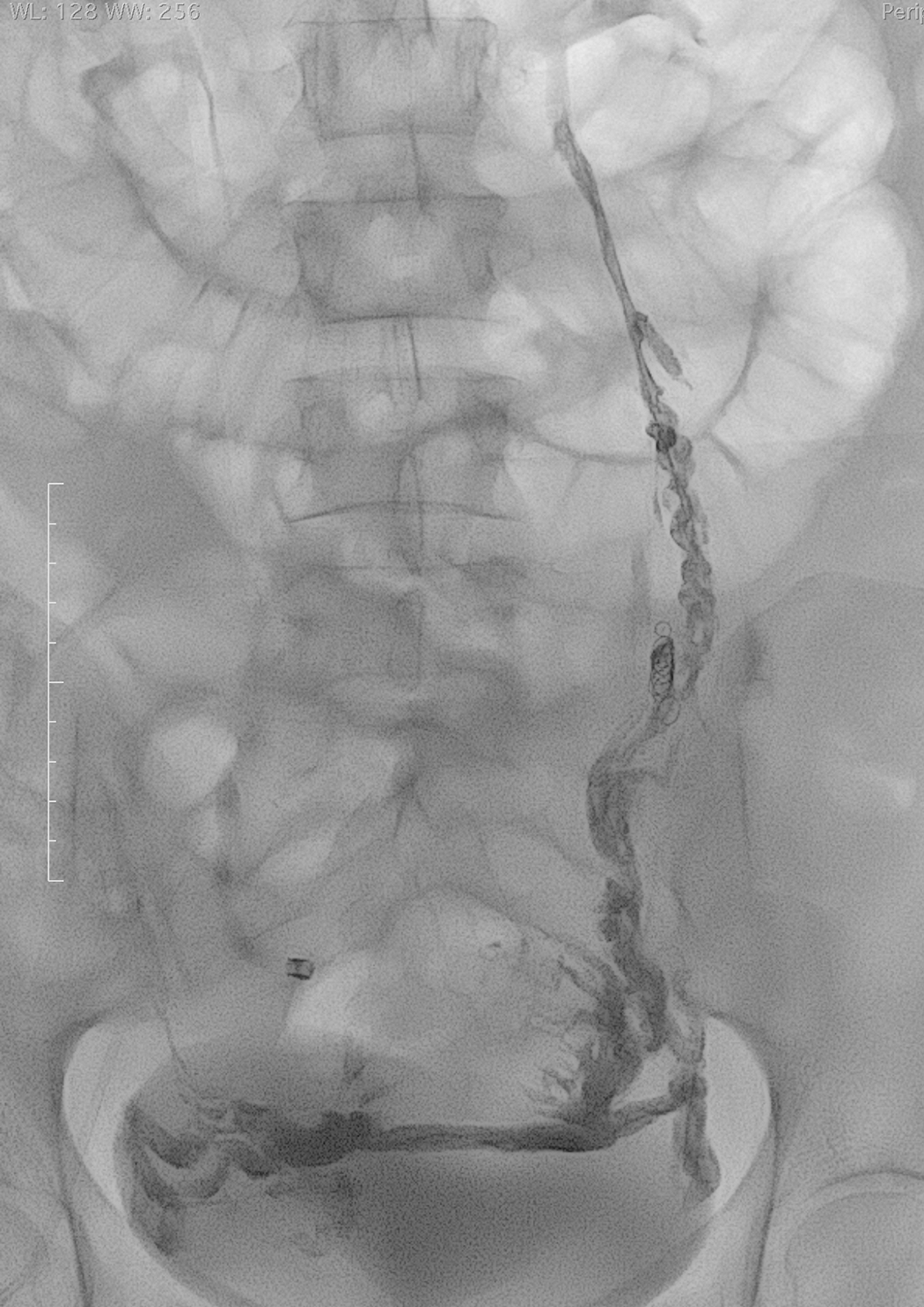

Supplement: Supplementary Figure 1 — Onyx® embolization for recurrence of pelvic congestion syndrome (PCS). Patient referred to our institution for recurrence of PCS. The treatment had been performed in two stages in two different centers 3 years earlier. A first embolization of the uterovaginal varices and the left ovarian vein with coils and aetoxisclerol. The second embolization of uterovaginal, right uterovaginal, and a recurrence of the left ovarian vein with cyanoacrylate glue. (A) Venography demonstrated a recurrence of reflux of the left ovarian vein with numerous collaterals around the coils. This recurrence is responsible for pelvic venous insufficiency with ovarian varicose veins, dilated left uterine, and utero-ovarian plexuses with contralateral communication by dilated uterine and arcuate veins supplying the right uterine plexus, which is also dilated. (B) Valsalva maneuver shows bilateral uterovaginal reflux. (C) Onyx® embolization. (D) Non-subtracted pelvic venography shows complete occlusion with Onyx® in dilated left ovarian veins, left dilated utero-ovarian plexus, bilateral uterovaginal plexus, and arcuate uterine veins. *Black, Cyanoacrylate glue; *White, Coils; Arrows, Dilated veins of the utero-ovarian plexus; Double arrows, Dilated veins of the uterovaginal plexus; Arrows head, Onyx® Double arrows head, Microcatheter. [file Image_1.tif]

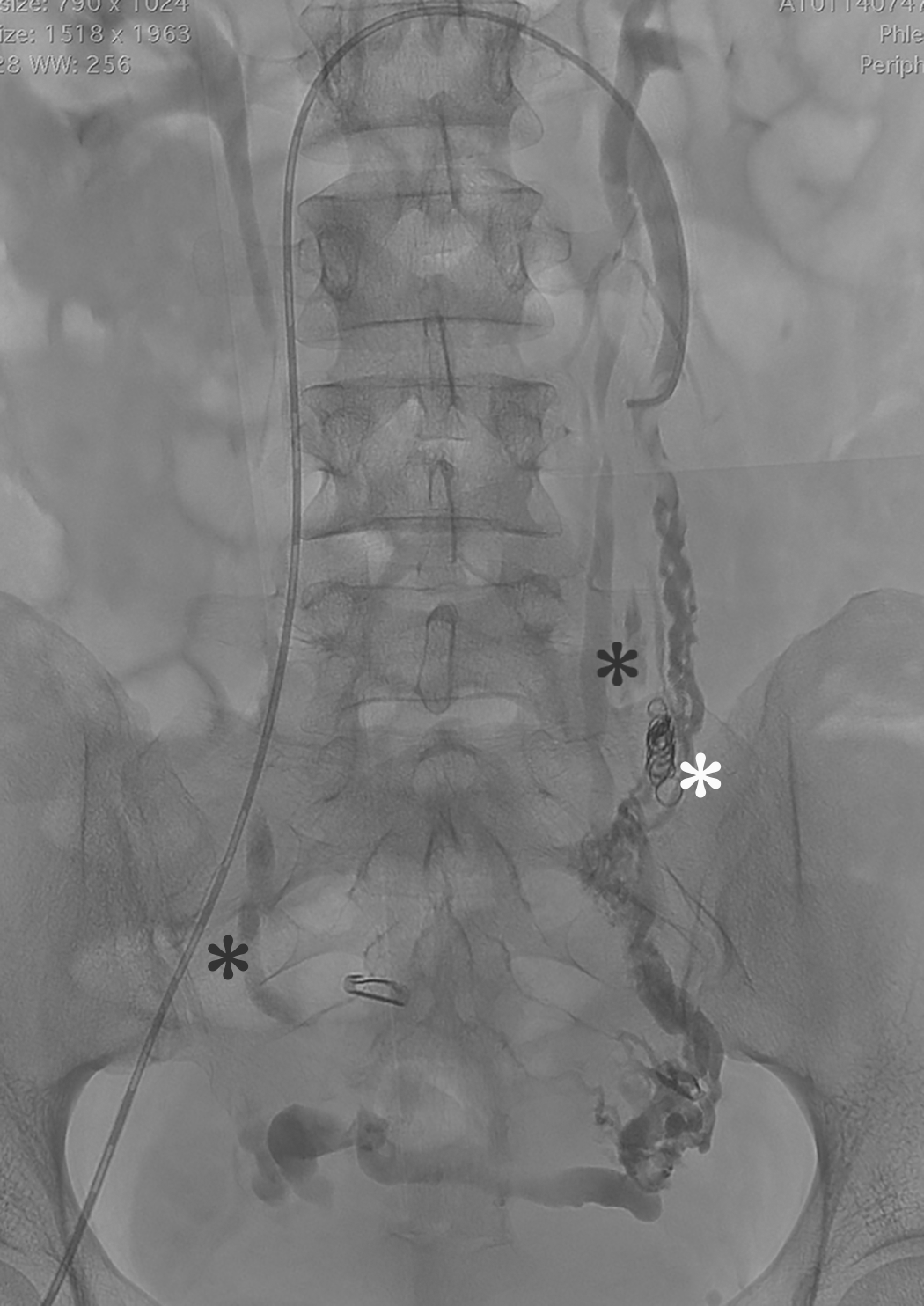

Supplement: Supplementary file 2 [file Image_2.tif]

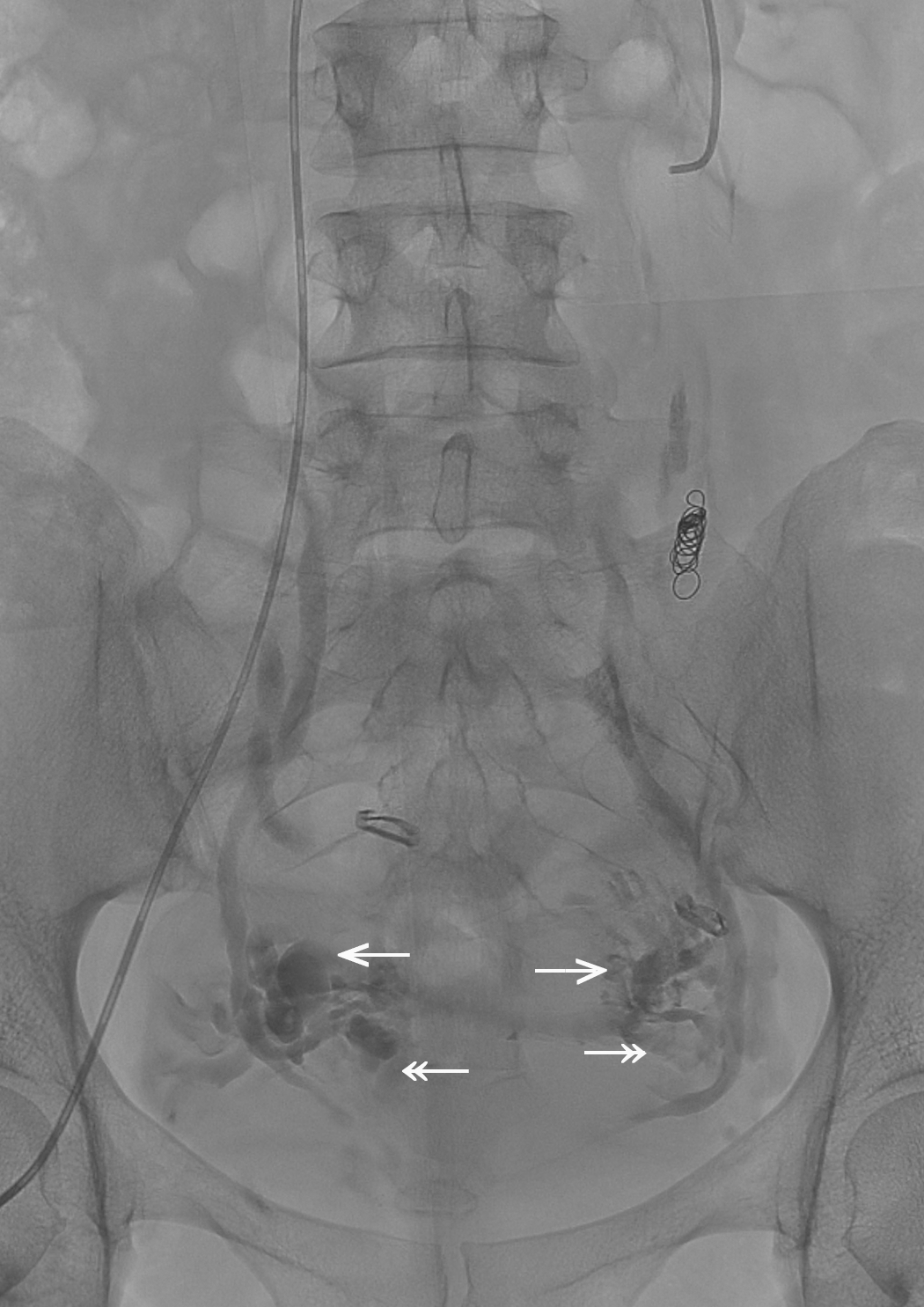

Supplement: Supplementary file 3 [file Image_3.tif]

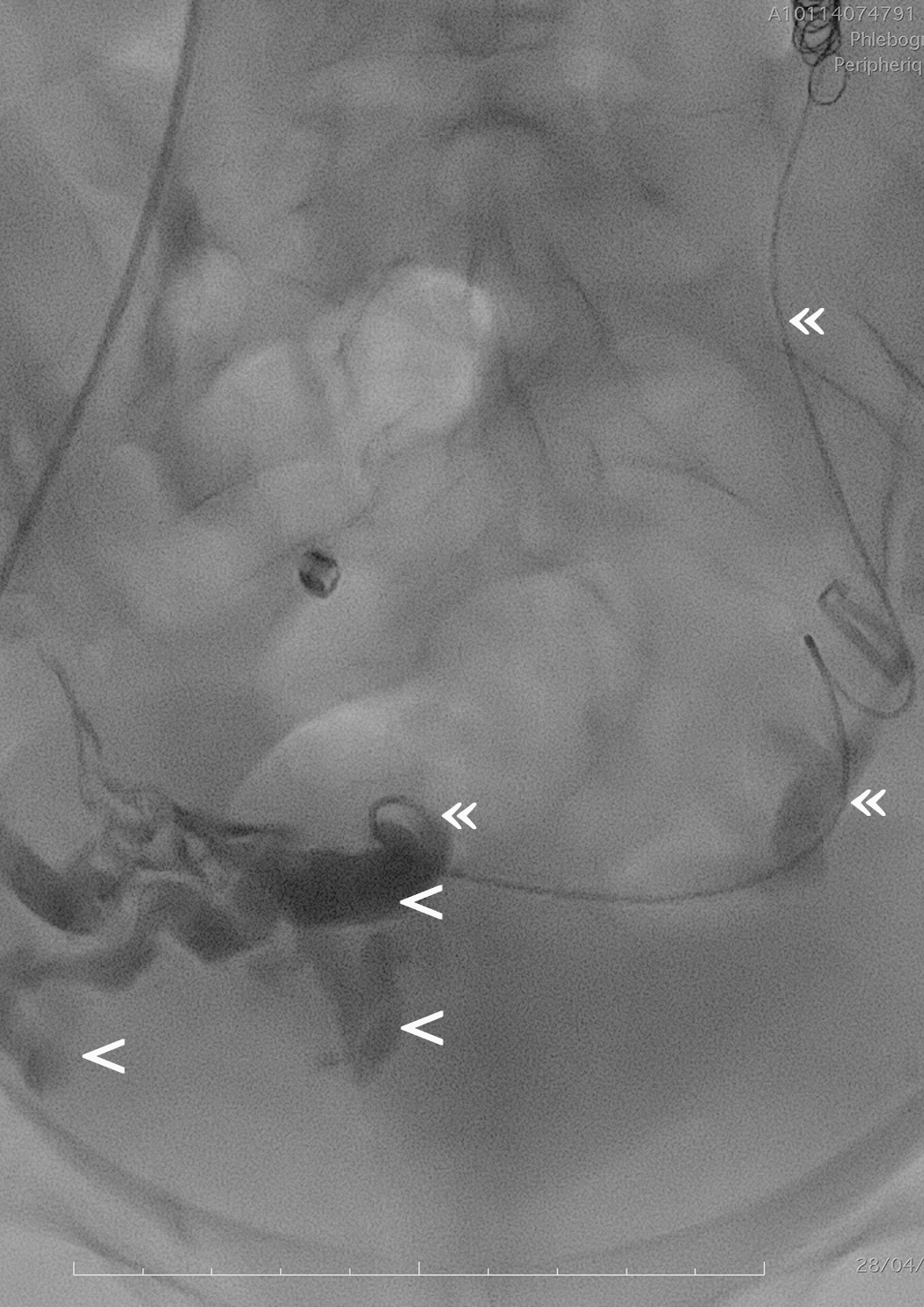

Supplement: Supplementary file 4 [file Image_4.tif]
